# ATP Released by Injured Neurons Activates Schwann Cells

**DOI:** 10.3389/fncel.2016.00134

**Published:** 2016-05-23

**Authors:** Samuele Negro, Elisanna Bergamin, Umberto Rodella, Elisa Duregotti, Michele Scorzeto, Kees Jalink, Cesare Montecucco, Michela Rigoni

**Affiliations:** ^1^Department of Biomedical Sciences, University of PadovaPadua, Italy; ^2^Division of Cell Biology, The Netherlands Cancer InstituteAmsterdam, Netherlands; ^3^National Research Council, Institute of NeurosciencePadua, Italy

**Keywords:** primary neurons, Schwann cells, ATP, calcium, cAMP, ERK 1/2, CREB, apyrase

## Abstract

Injured nerve terminals of neuromuscular junctions (NMJs) can regenerate. This remarkable and complex response is governed by molecular signals that are exchanged among the cellular components of this synapse: motor axon nerve terminal (MAT), perisynaptic Schwann cells (PSCs), and muscle fiber. The nature of signals that govern MAT regeneration is ill-known. In the present study the spider toxin α-latrotoxin has been used as tool to investigate the mechanisms underlying peripheral neuroregeneration. Indeed this neurotoxin induces an acute, specific, localized and fully reversible damage of the presynaptic nerve terminal, and its action mimics the cascade of events that leads to nerve terminal degeneration in injured patients and in many neurodegenerative conditions. Here we provide evidence of an early release by degenerating neurons of adenosine triphosphate as *alarm* messenger, that contributes to the activation of a series of intracellular pathways within Schwann cells that are crucial for nerve regeneration: Ca^2+^, cAMP, ERK1/2, and CREB. These results contribute to define the cross-talk taking place among degenerating nerve terminals and PSCs, involved in the functional recovery of the NMJ.

## Introduction

The neuromuscular junction (NMJ) is the specialized anatomical structure where the electric signal traveling along the axon is converted into a chemical message, which binds to post-synaptic receptors causing muscle contraction. This synapse consists of three main components: motor axon terminal (MAT), muscle fiber (MF), and perisynaptic Schwann cells (PSCs). MAT is covered by a multi-cellular carpet of PSCs, and MAT–PSCs are enveloped by the permeable basal lamina (BL) separating axon terminal from MF.

Neuromuscular junctions are often exposed to mechanical traumas and represent the main target of several chemicals and biologic toxins. Indeed, during evolution, both animals and bacteria have developed toxins which selectively interfere with nerve–muscle transmission, causing a neuroparalysis which frequently leads to death (Schiavo et al., 2000). Moreover, in many neuromuscular diseases such as amyotrophic lateral sclerosis and immune-mediated disorders, including the Guillain–Barré and Miller Fisher syndromes, the synaptic transmission between motor neurons (MNs) and muscle cells is compromised, with demyelination and axonal degeneration (Yuki and Hartung, 2012; Moloney et al., 2014).

For these reasons and for its essential role in life and survival the NMJ, at variance from most mammalian tissues, has retained through evolution the capacity to regenerate (Brosius Lutz and Barres, 2014). PSCs are main players in the regeneration process: following injury they de-differentiate and acquire phagocytic properties, remove cellular debris and guide axon growth to its original site with recovery of function (Sanes and Lichtman, 1999; Jessen et al., 2015). Together with MF, PSCs produce and release a series of factors that act on the stump of MAT inducing its regrowth toward its original position, wherefrom it resumes the regulated release of neurotransmitter (Son et al., 1996; Sanes and Lichtman, 1999; Darabid et al., 2014). This complex response is governed by molecular signals that are exchanged among the three cellular components of the NMJ and the BL, whose nature is largely unknown.

To study the cross-talk among the different components of the NMJ during nerve degeneration and regeneration, we have recently set up an innovative experimental system, based on the use of animal presynaptic neurotoxins that cause a rapid and reversible degeneration confined to the sole MAT without inflammation (Duregotti et al., 2015). More specifically, here we have used α-latrotoxin (α-Ltx), a pore-forming toxin from the venom of the spider *Latrodectus mactans*, whose envenomation causes the neuroparalysis of peripheral skeletal muscles and of autonomic nerve terminals. α-Ltx induces a very rapid nerve terminal blockade by forming transmembrane ion channels with consequent massive Ca^2+^entry, exocytosis of synaptic vesicles and mitochondrial damage (Hurlbut and Ceccarelli, 1979; Ceccarelli and Hurlbut, 1980; Rosenthal et al., 1990; Südhof, 2001; Ushkaryov et al., 2008). This leads to a Ca^2+^-induced degeneration of MAT limited to the unmyelinated endplate; strikingly, a complete recovery of NMJ function is achieved within few days in mice, weeks in humans (Duchen et al., 1981; Kularatne and Senanayake, 2014). Given the high reproducibility of the process, using this experimental approach, we have recently demonstrated that α-Ltx-treated primary neurons release signaling molecules derived from mitochondria: hydrogen peroxide, mitochondrial DNA, and cytochrome c, which activate isolated primary SCs, SCs co-cultured with neurons and *in vivo* at the NMJ through the MAPK pathway (Duregotti et al., 2015).

In search of additional candidate signaling molecules that once released by degenerating neurons might stimulate PSCs, we focused our attention on adenosine triphosphate (ATP). Beside its known role as energy source, ATP is also an extracellular messenger acting on different types of purinergic receptors. ATP is an important signaling molecule in the peripheral nervous system (PNS), where it plays a crucial role in chemical communication between several cell types, and can also act as growth and trophic factor by regulating calcium and cyclic AMP (cAMP) signaling in target cells (Fields and Burnstock, 2006). A first evidence indicating that neurons use ATP to communicate with glial cells was obtained at the frog NMJ (Robitaille, 1995; Rochon et al., 2001). During synaptic activity ATP is co-released with acetylcholine (Ach) from nerve endings, and evokes calcium responses in PSCs by activating type 2 purinergic receptors (Robitaille, 1995). Thus, through ATP- and Ach-sensing PSCs are able to detect and monitor synaptic activity, and, indirectly, synaptic integrity. We wondered whether SCs could be activated by ATP released by injured nerve terminals. Moreover, we investigated which downstream signaling pathways are activated in these cells.

## Materials and Methods

α-Latrotoxin was purchased from Alomone (Israel). The purity of the toxin was checked by SDS/PAGE, and its neurotoxicity was tested in the *ex vivo* mouse nerve-hemidiaphragm preparation, as previously described (Rigoni et al., 2005).

Unless stated otherwise reagents were purchased from Sigma.

### Primary Cell Cultures

Experiments on Wistar rats (Plaisant Srl) were performed in accordance with the Council Directive 2010/63/EU of the European Parliament, the Council of September 22, 2010 on the protection of animals used for scientific purposes, approved by the local committee and by the Italian Ministry of Health.

Primary cultures of rat cerebellar granular neurons (CGNs), spinal motor neurons, SCs, and their co-cultures were described previously (Rigoni et al., 2004; Duregotti et al., 2015).

### ATP Measurements

Adenosine triphosphate was quantified in the supernatant of primary neurons exposed for different time periods to α-Ltx using the commercial ATP Lite One-Step kit (Perkin–Elmer). Quick centrifugation of the plates was performed to eliminate cell debris. Luminescence was measured with a luminometer (Infinite M200 PRO, Tecan), and ATP concentration determined using a standard curve.

### Calcium Imaging

Isolated SCs or co-cultures with primary neurons were loaded for 10 min with the calcium indicator Fluo-4AM (4 μM, Invitrogen). After loading, cells were washed and then moved to the stage of an inverted fluorescence microscope (Eclipse-Ti; Nikon Instruments) equipped with the perfect focus system (PFS; Nikon Instruments) and with high numerical aperture oil immersion objectives (60×). Calcium signals were recorded in control samples or in samples exposed to α-Ltx 0.1 nM with excitation of the fluorophore performed at 465–495 by means of an Hg arc lamp (100 W; Nikon). Emitted fluorescence was collected at 515–555 nm. Fluorescence (F) was measured in a selected region of interest (ROI) containing cell cytosol and corrected for background. Measurements were expressed as *F*/*F*_0_ ratio, where *F*_0_ represents the fluorescence level at *t* = 0. Images were acquired for 10 or 40 min every 20 s. In some experiments apyrase (1.5 U/ml) was added 5 min before intoxication, and left throughout.

### Cyclic AMP Detection

A fourth generation of *Epac-based* fluorescence resonance energy transfer (FRET) probe for cAMP detection was used. This sensor consists of the cAMP-binding protein EPAC sandwiched between mTurquoise2, a very bright- and bleaching-resistant donor fluorescent protein, and a novel acceptor cassette consisting of a tandem of two Venus fluorophores (Klarenbeek et al., 2015). Briefly, SCs alone or in co-cultures with neurons were transfected with 1 μg of the probe with Lipofectamine 2000 (Life Technologies). Experiments were performed 24 h after transfection. Cells were monitored using an inverted fluorescence microscope (Eclipse-Ti; Nikon Instruments) equipped with the PFS (Nikon Instruments). Excitation of the fluorophore was performed by an Hg arc lamp (100 W; Nikon) using a 435-nm filter (10-nm bandwidth). Yellow fluorescent protein (YFP) and cyan fluorescent protein (CFP) intensities were recorded with a cooled CCD camera (C9100-13; Hamamatsu) equipped with a 515-nm dichroic mirror at 530 nm (25-nm bandwidth) and 470 nm (20-nm bandwidth), respectively. Signals were digitized and FRET was expressed as the ratio between donor and acceptor signals. YFP and CFP intensities were corrected for background. After 3 min recordings isolated SCs were exposed to ATP (50 μM), and co-cultures were incubated with α-Ltx (0,1 nM); a final stimulation with 25 μM forskolin was performed at the end of each experiment to maximally raise cAMP levels. In some experiments apyrase (1.5 U/ml) was added 5 min before intoxication, and left throughout.

### Western Blotting

Following treatments samples were lysed in lysis buffer (Hepes 10 mM, NaCl 150 mM, SDS 1%, EDTA 4 mM, protease inhibitors cocktail – Roche -, and phosphatase inhibitor cocktail). Seven to ten micrograms of total lysates from SCs or co-cultures were loaded on Precast 4–12% SDS-polyacrylamide gels (Life Technologies) and transferred onto nitrocellulose paper in a refrigerated chamber. Protein concentration was quantified using the BCA assay (Protein Assay Kit, Pierce, MO, USA). After saturation, membranes were incubated o/n with a rabbit polyclonal antibody for phospho-p44/42 MAPK (p-ERK 1/2, 1:1000, Cell Signaling), or with a rabbit polyclonal against phospho-CREB (Cell Signaling, 1:1000), followed by a secondary anti-rabbit secondary antibody HRP-conjugated (Life Technologies, 1:2000). Chemiluminescence was developed with the Luminata^TM^ Crescendo (Millipore) or ECL Advance Western blotting detection system (GE Healthcare), and emission measured with ChemiDoc XRS (Bio-Rad). For densitometric quantification, the bands of interest were normalized to the housekeeping protein Hsc70 (mouse monoclonal, 1:10000, Synaptic Systems). Band intensities were quantified on the original files with the software Quantity One (Bio-Rad). None of the bands reached signal saturation. In some experiments apyrase (1.5 U/ml) was added 5 min before intoxication, and left throughout.

### Immunofluorescence

Primary SCs were processed for immunofluorescence as described in Duregotti et al. (2015). The following primary antibodies were used: rabbit polyclonal for phospho-p44/42 MAPK (p-ERK 1/2, 1:500, Cell Signaling), rabbit polyclonal against phospho-CREB (Cell Signaling, 1:800), mouse monoclonal for S100 (Sigma, 1:200). Secondary antibodies Alexa-conjugated (Life Technologies, 1:200) were employed.

### Statistical Analysis

The sample size (*N*) of each experimental group is described in each corresponding figure legend; at least three biological replicates were performed. GraphPad Prism software was used for all statistical analyses. Quantitative data displayed as histograms are expressed as means ± SEM (represented as error bars). Results from each group were averaged and used to calculate descriptive statistics. Significance was calculated by Student’s *t*-test (unpaired, two-side). *P*-values less than 0.05 were considered significant.

## Results

### ATP Is Released by Degenerating Neurons

**FIGURE 1:**
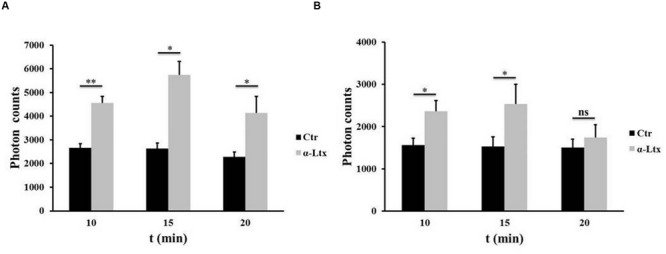
**Adenosine triphosphate (ATP) is released by degenerating neurons.** Time-course of ATP release by cerebellar granular neurons (CGNs; **A**) and spinal cord motor neurons (SCMNs; **B**) exposed to α-Ltx for 20 min compared to control neurons. The release is expressed as photon counts. ^∗^*P* < 0.05; ^∗∗^*P* < 0.01. *N* = 5 (Student’s *t*-test, unpaired, two-side); ns = not significant.

Cerebellar granular neurons and spinal cord motor neurons (SCMNs) exposed to nanomolar concentrations of α-Ltx progressively release ATP in the supernatant, measured by a luminometric assay, as shown in **Figure 1**. Maximum release takes place within 15 min of intoxication. Under the same experimental conditions no changes in plasma membrane permeability take place, as shown previously by the lack of lactate dehydrogenase activity in the cell supernatant and by calcein retention, indicating that ATP is not released merely as a consequence of cell lysis (Duregotti et al., 2015).

### Neuronal ATP Triggers Calcium Spikes in Schwann Cells

**FIGURE 2:**
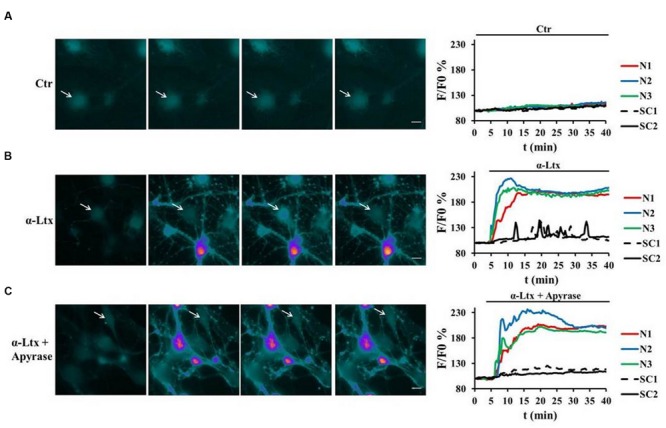
**Neuronal ATP triggers calcium spikes in Schwann cells co-cultured with degenerating neurons.** Co-cultures of primary SCs and CGNs loaded with Fluo4 AM were exposed to α-Ltx; intracellular calcium changes are represented in a pseudocolor scale (*blue*: low concentration; *white*: high concentration), and quantified. **(A)** In controls no calcium increase was detected either in neurons (N, colored lines) or in SCs (SC, black lines) during 40 min incubation. **(B)** In intoxicated co-cultures a rapid and progressive calcium increase was detected in neurons (*N*, colored lines), followed by calcium spikes in SCs (arrows, black lines). **(C)** Apyrase preincubation nearly abolishes calcium spikes in SCs, leaving calcium increase in neurites unaffected. Representative traces are reported *N* = 5.

As terminal SCs express on their surface different types of purinergic receptors which activate various intracellular pathways, we examined whether SCs can be a target of ATP, and which signaling pathways could be thereby activated. Indeed, primary SCs loaded with the calcium indicator Fluo4 AM respond to micromolar ATP with a peak of calcium (Supplementary Figure S1B, pseudocolor images and quantification). When CGNs in co-cultures with SCs are exposed to α-Ltx, *bulges* or varicosities appear along neurites within few minutes, and intracellular calcium levels progressively rise within these characteristic rounded structures, hallmarks of intoxication, and along neurites (Bonanomi et al., 2005; Tedesco et al., 2009). Immediately after, calcium spikes are observed in SCs (**Figure 2B**). Pre-incubation with apyrase, which hydrolyses ATP to AMP and inorganic phosphate, strongly reduces calcium spikes in co-cultured SCs, leaving neuronal calcium levels unaffected (**Figure 2C**). No calcium changes are observed under control conditions (**Figure 2A**; Supplementary Figure S1A).

### Neuronal ATP Triggers cAMP Production in Schwann Cells

**FIGURE 3:**
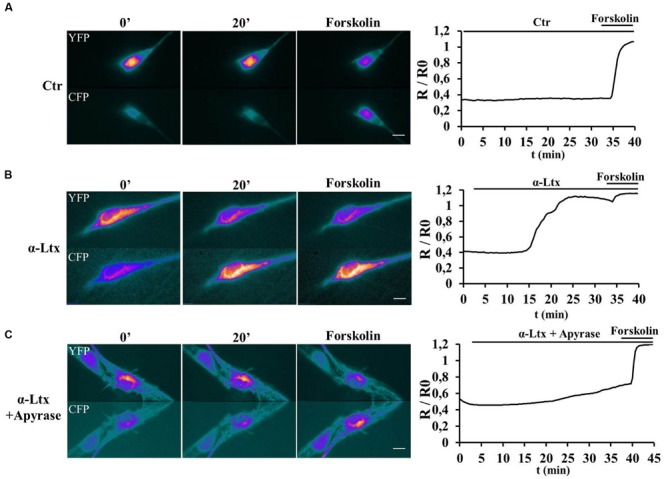
**Neuronal ATP triggers cyclic AMP (cAMP) production in Schwann cells co-cultured with degenerating neurons.** SCs in co-cultures were transfected with the H187 sensor and fluorescence resonance energy transfer (FRET) measured under control conditions **(A)** or during exposure to α-Ltx plus/minus apyrase (**B** and **C**, respectively, toxin added at *t* = 3 min). Forskolin was added at the end of the experiment as positive control. During FRET a decrease in Yellow fluorescent protein (YFP) fluorescence and a parallel increase in the cyan fluorescent protein (CFP) one take place, as indicated by the pseudocolor images (*blue*: low fluorescence; *white*: high fluorescence). Quantification is shown in the right panels, where FRET (*R*/*R*_0_) is expressed as the ratio between the donor and the acceptor signals (*R*) corrected for the background (*R*_0_). In controls no cAMP increase is detected in SCs **(A)**, whereas a progressive rise is observed in co-cultures where neurons are exposed to the neurotoxin **(B)**. **(C)** Pretreatment with apyrase strongly reduces cAMP levels in SCs.

Purinergic receptors transduce the extracellular input ATP also via activating cAMP signaling (Fields and Burnstock, 2006). SCs transfected with a new generation *Epac* probe and *live imaged* as described (Klarenbeek et al., 2015) respond to exogenous ATP by raising their cAMP content (Supplementary Figure S2B). No FRET was measured in controls (Supplementary Figure S2A). We next imaged cAMP levels in SCs co-cultured with CGNs before and after exposure to α-Ltx (**Figure 3**). cAMP levels remain constant in controls (**Figure 3A**). By 15 min incubation with α-Ltx cAMP progressively increases in SCs, then reaching a plateau. The addition of forskolin at the end of the experiment causes a little further increase (**Figure 3B**). Cyclic-AMP rise is at least in part triggered by neuronal ATP, since a reduction is observed upon preincubation with apyrase (**Figure 3C**). Thus, neuronal ATP released during degeneration contributes to cAMP generation in nearby SCs. The partial effect of apyrase is expected since other mediators released by intoxicated neurons are likely to contribute to cAMP signaling.

### Neuronal ATP Induces ERK 1/2 and CREB Phosphorylation in Schwann Cells

**FIGURE 4:**
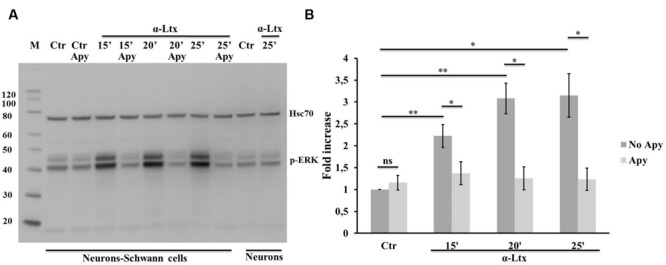
**Neuronal ATP induces ERK 1/2 phosphorylation in Schwann cells co-cultured with degenerating neurons.** Time-course of ERK 1/2 activation in co-cultures exposed for 25 min to α-Ltx in the presence or absence of apyrase. **(A)** Representative Western blot showing a sustained phospho-ERK increase in co-cultures exposed to α-Ltx, and its reduction by apyrase treatment. No phospho-ERK is detected in isolated neurons exposed to the toxin, demonstrating that phospho-ERK 1/2 signal in co-cultures lysates derives from SCs. **(B)** For the quantification data were normalized for the housekeeping Hsp70 and expressed as fold increase with respect to control. ^∗^*P* < 0.05; ^∗∗^*P* < 0.01. *N* = 3 (Student’s *t*-test, unpaired, two-side); ns = not significant.

The MAPK signaling pathway plays a central role in controlling SCs plasticity and peripheral nerve regeneration via the activation of ERK 1/2 and JNK (Arthur-Farraj et al., 2012; Napoli et al., 2012). We have recently reported that several mitochondrial *alarmins* released by degenerating neurons activate the ERK 1/2 pathway in SCs (Duregotti et al., 2015). Therefore, ATP was tested as one possible activator of the MAP kinase pathway within SCs in neuron-SC co-cultures exposed to α-Ltx. A control experiment was performed with isolated SCs which respond to ATP by phosphorylating ERK 1/2 very rapidly (Supplementary Figure S3A). Phospho-ERK signal has a cytoplasmic localization after 5 min incubation, nuclear at 10 min (Supplementary Figure S3B). **Figure 4** shows the sustained ERK 1/2 phosphorylation in SCs in co-cultures with CGNs exposed to α-Ltx and the relative quantification. Phospho-ERK levels are reduced by apyrase, thus indicating that ATP released from degenerating neurons participate in the induction of the MAP kinase signaling pathway in co-cultured SCs. This pathway is not activated in isolated neurons exposed to the sole toxin.

**FIGURE 5:**
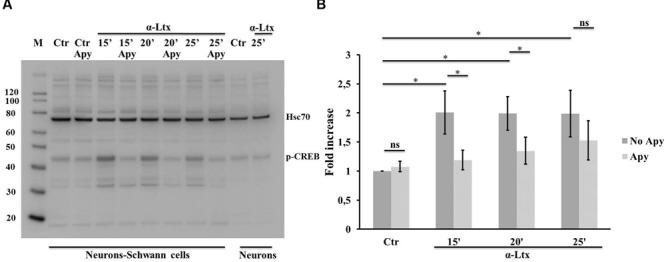
**Neuronal ATP induces CREB phosphorylation in Schwann cells co-cultured with degenerating neurons.** Time-course of CREB phosphorylation in co-cultures exposed for 25 min to α-Ltx in the presence or absence of apyrase. **(A)** Representative Western blot showing phospho-CREB increase in co-cultures exposed to α-Ltx, and its reduction by apyrase treatment. No phospho-CREB is detected in isolated neurons exposed to the toxin, demonstrating that phospho-CREB signal in co-cultures lysates derives from SCs. **(B)** For the quantification data are normalized for the housekeeping Hsp70 and expressed as fold increase with respect to control. ^∗^*P* < 0.05. *N* = 4 (Student’s *t*-test, unpaired, two-side); ns = not significant.

Activation of both ERK 1/2 and cAMP pathways are known to promote the transcriptional activity of CREB, one of the best understood phosphorylation-dependent transcription factors, involved in a variey of cellular processes and in neuron-glia communication (Tabernero et al., 1998). Similarly to ERK, also CREB becomes phosphorylated in isolated SCs exposed to ATP (Supplementary Figures S3C,D), and in co-cultures with CGNs upon α-Ltx treatment (**Figure 5**), and the extent of phosphorylation is reduced by apyrase.

## Discussion

Perisynaptic Schwann cells are specialized glial cells that tightly surround the NMJ and actively participate in its maintenance and repair. It is believed that at the NMJ an intense cross-talk takes place under physiological and pathological conditions among its components: MAT, PSCs, MF, and BL.

In the present study, we have investigated the putative role of ATP as *alarm* molecule involved in the intercellular signaling among injured neurons and SCs. Our data provide evidence of an early release of ATP by neurons exposed to a presynaptic neurotoxin that induces degeneration of nerve terminals. It is generally assumed that the main source of ATP acting on purinoceptors are dying cells, but this is not the case at least in the present mouse model of peripheral neurodegeneration, since plasma membrane integrity is well preserved at the time points of ATP release.

We next investigated which downstream signaling pathways are activated in SCs by nerve degeneration. In fact, ATP signals through purinergic receptors, and glial cells express a range of these receptors, whose activation can elicit different signaling pathways within the cell, including Ca^2+^, cAMP, inositol-1,4,5-triphosphate, phospholipase C, and additional ones (Fields and Burnstock, 2006). We found both Ca^2+^ and cAMP increase within SCs during intoxication, with kinetics that well correlate with that of ATP release. Calcium spikes in SCs in co-cultures with neurons follow α-Ltx-induced calcium increases within neuronal *bulges*, that are sites of stimulated exocytosis and unbalanced endocytosis (Tedesco et al., 2009). ATP contributes to calcium rise in SCs (together with other *alarmins* of neuronal origin), as the latter is reduced by apyrase pretreatment.

Also cAMP increases in SCs co-cultured with neurons exposed to α-Ltx, as measured by a new generation FRET sensor, and this is dependent, at least in part, on neuronal ATP. After PNS injury, SCs undergo a *transdifferentiation* process: they dedifferentiate, proliferate, and then differentiate back to a myelinating phenotype (Jessen et al., 2015; Jessen and Mirsky, 2016). The transition between these stages relies heavily on cAMP signaling (Knott et al., 2014). This process is less well defined within PSCs, which are clearly different from their myelinating counterparts. However, given that cAMP is implicated as an important second messenger regulating phagocytosis (Pryzwansky et al., 1998), it is likely that its signaling cascade could be important also for PSCs that display macrophagic-like properties during nerve regeneration (Reichert et al., 1994; Duregotti et al., 2015).

In addition, we have found here that ATP contributes to ERK 1/2 activation within SCs co-cultured with degenerating neurons, since pretreatment with apyrase lowers phospho-ERK levels. This observation is in keeping with the fact that MAPK signaling plays a central role in controlling SC plasticity and peripheral nerve regeneration (Arthur-Farraj et al., 2012; Napoli et al., 2012), and with our recent report that a major trigger of ERK 1/2 phosphorylation in SCs is hydrogen peroxide, that is produced inside disfunctional mitochondria during MAT degeneration (Duregotti et al., 2015).

Both MAPK and cAMP-dependent protein kinase A engagement can initiate the transcription of genes containing a cAMP-responsive element, under the control of CREB transcriptional activity, in response to a vast array of stimuli including neurotransmitters, hormones, growth factors, synaptic activity, stressors, and inflammatory cytokines (Shaywitz and Greenberg, 1999). Stewart (1995) first reported high levels of phospho-CREB *in situ* in SCs throughout nerve development and after nerve transection. Indeed we found CREB phosphorylated in our co-culture system following neurotoxin exposure: phospho-CREB is detectable at early time points during intoxication, and ATP hydrolysis lowers its levels.

## Conclusion

We have shown here that ATP is rapidly released by injured neurons, and it contributes to the activation of a series of intracellular signaling pathways in SCs including Ca^2+^, adenylate cyclase, ERK 1/2, and CREB, that are expected to drive the complex response of these glial cells functional to the recovery of nerve function. We believe that the present study will help to define the intercellular cross-talk that takes plays at the NMJ not only during the poisoning by a spider toxin, but that could be extended to different forms of neurodegeneration affecting the peripheral presynaptic nerve terminals.

## Author Contributions

The study was designed by MR and CM. SN, MS, UR, ED, and EB performed and analyzed experiments. KJ provided reagents. SN, MR, and CM prepared the manuscript.

## Conflict of Interest Statement

The authors declare that the research was conducted in the absence of any commercial or financial relationships that could be construed as a potential conflict of interest.
