# Supplementary figures and images for: ATP Released by Injured Neurons Activates Schwann Cells
Source: Front Cell Neurosci. 2016 May 23;10:134. doi: 10.3389/fncel.2016.00134 (PMC4876115; doi:10.3389/fncel.2016.00134)

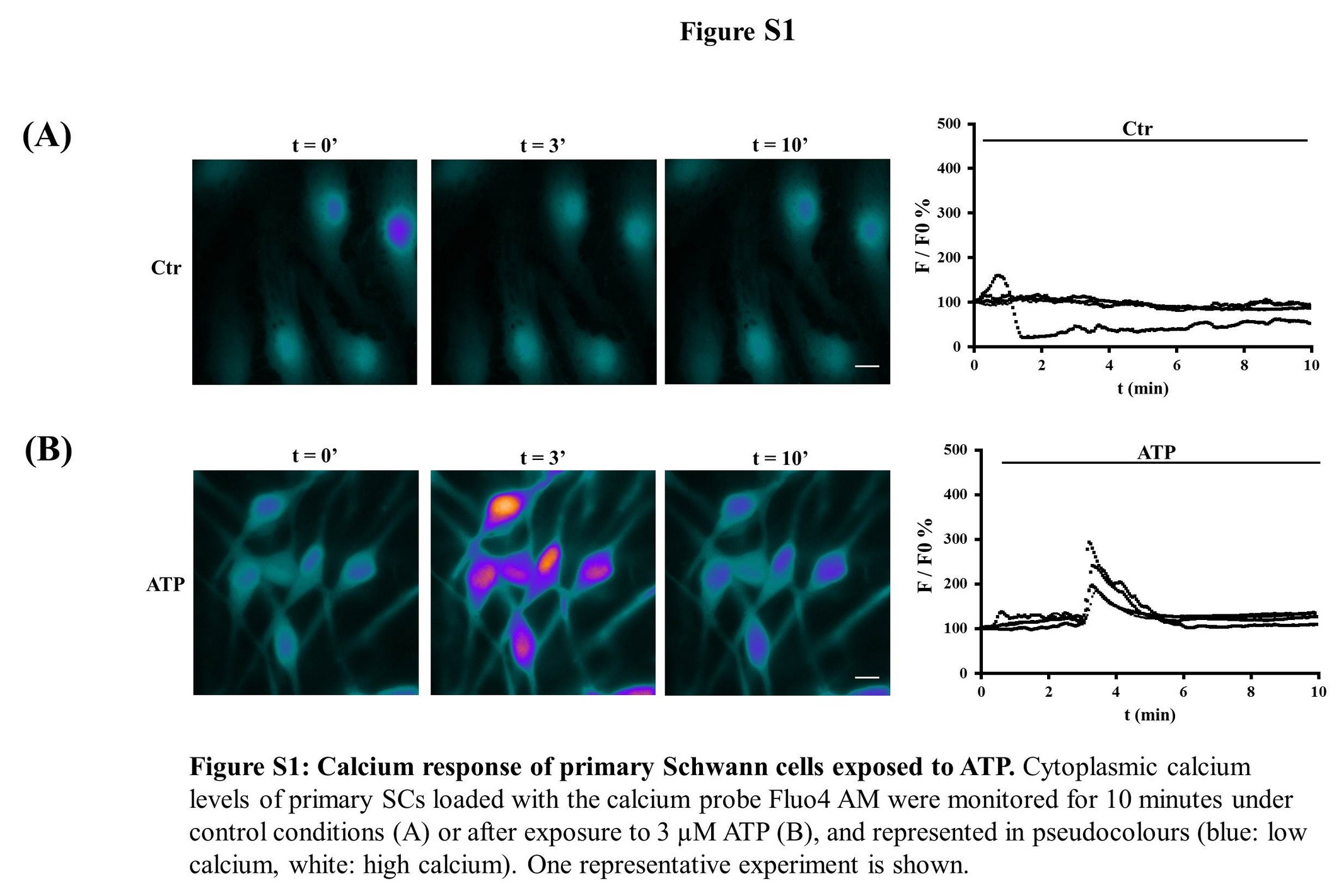

Supplement: Supplementary file 1 [file Image_1.JPEG]

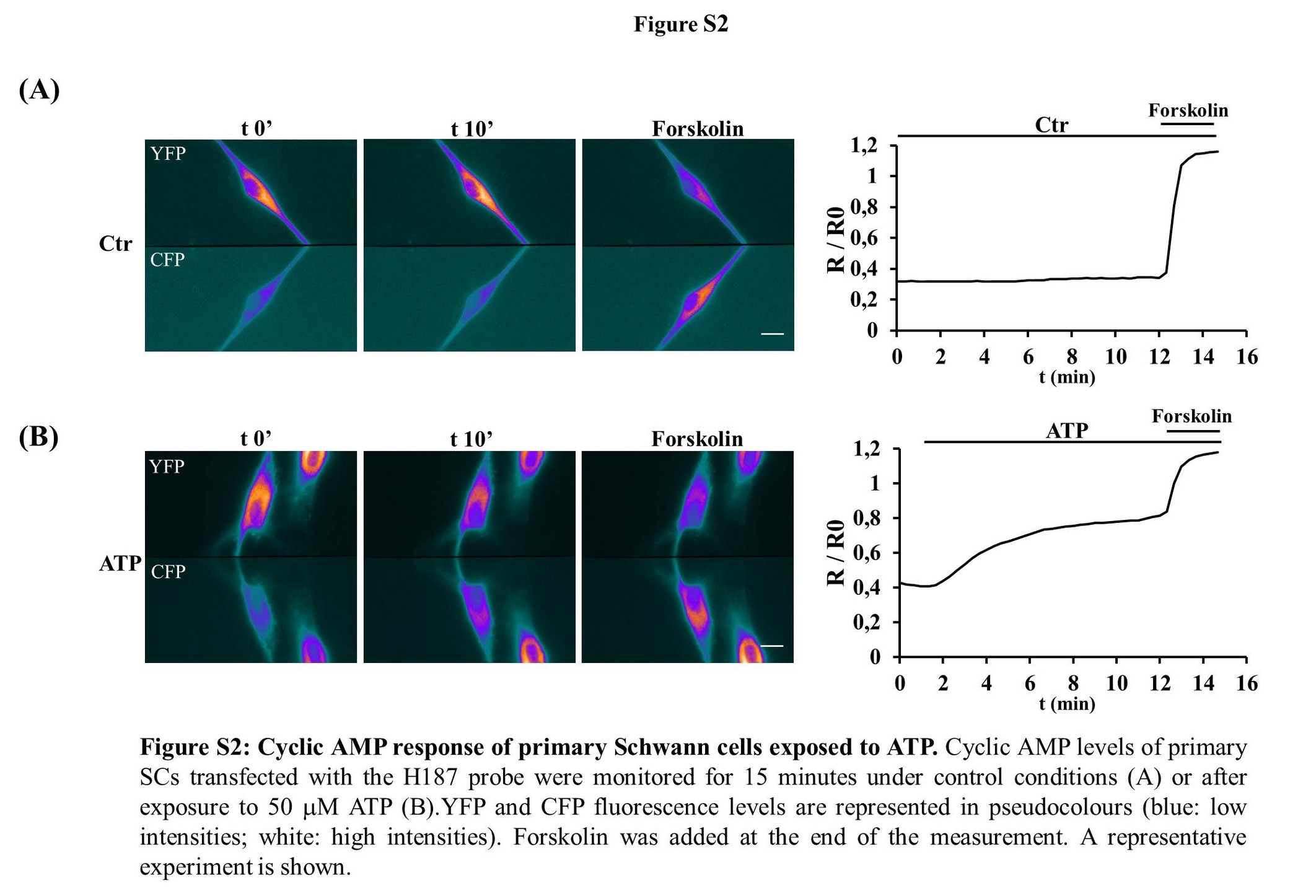

Supplement: Supplementary file 2 [file Image_2.JPEG]

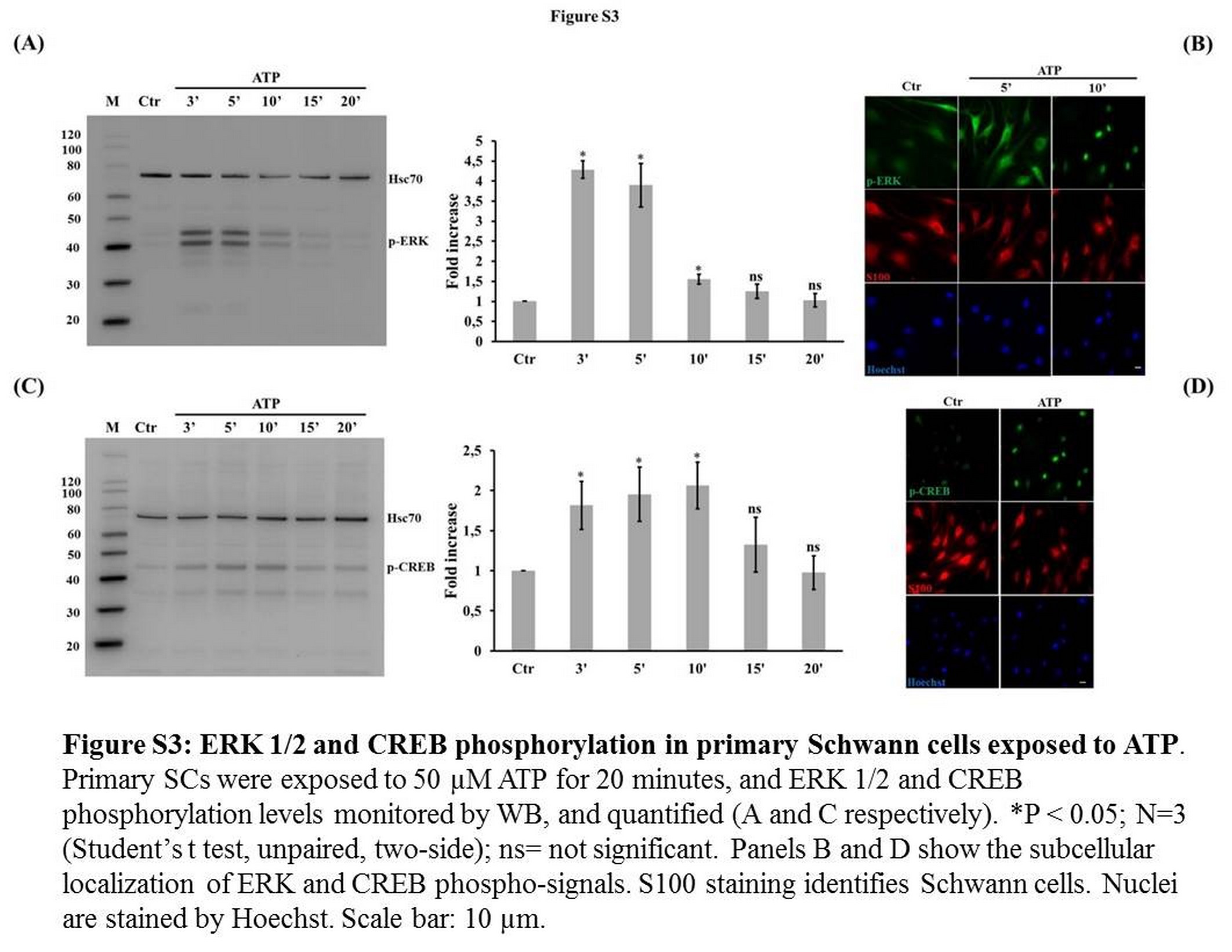

Supplement: Supplementary file 3 [file Image_3.JPEG]
